# Blood Contamination in Saliva: Impact on the Measurement of Salivary Oxidative Stress Markers

**DOI:** 10.1155/2015/479251

**Published:** 2015-08-11

**Authors:** Natália Kamodyová, Lenka Baňasová, Katarína Janšáková, Ivana Koborová, Ľubomíra Tóthová, Peter Stanko, Peter Celec

**Affiliations:** ^1^Institute of Molecular Biomedicine, Faculty of Medicine, Comenius University, Sasinkova 4, 811 08 Bratislava, Slovakia; ^2^Department of Stomatology and Maxillofacial Surgery, Comenius University, Heydukova 10, 812 50 Bratislava, Slovakia; ^3^Center for Molecular Medicine, Slovak Academy of Sciences, Vlárska 7, 831 01 Bratislava, Slovakia; ^4^Institute of Pathophysiology, Faculty of Medicine, Comenius University, Sasinkova 4, 811 08 Bratislava, Slovakia; ^5^Department of Molecular Biology, Faculty of Natural Sciences, Comenius University, Mlynská Dolina, 842 15 Bratislava, Slovakia

## Abstract

Salivary oxidative stress markers represent a promising tool for monitoring of oral diseases. Saliva can often be contaminated by blood, especially in patients with periodontitis. The aim of our study was to examine the impact of blood contamination on the measurement of salivary oxidative stress markers. Saliva samples were collected from 10 healthy volunteers and were artificially contaminated with blood (final concentration 0.001–10%). Next, saliva was collected from 12 gingivitis and 10 control patients before and after dental hygiene treatment. Markers of oxidative stress were measured in all collected saliva samples. Advanced oxidation protein products (AOPP), advanced glycation end products (AGEs), and antioxidant status were changed in 1% blood-contaminated saliva. Salivary AOPP were increased in control and patients after dental treatment (by 45.7% and 34.1%, *p* < 0.01). Salivary AGEs were decreased in patients after microinjury (by 69.3%, *p* < 0.001). Salivary antioxidant status markers were decreased in both control and patients after dental treatment (*p* < 0.05 and *p* < 0.01). One % blood contamination biased concentrations of salivary oxidative stress markers. Saliva samples with 1% blood contamination are visibly discolored and can be excluded from analyses without any specific biochemic detection of blood constituents. Salivary markers of oxidative stress were significantly altered in blood-contaminated saliva in control and patients with gingivitis after dental hygiene treatment.

## 1. Introduction

Markers of oxidative stress in saliva have become an attractive tool for analyzing the pathogenesis and monitoring of oral and dental diseases. Cross-reacting substances in the mouth and saliva collection methods could influence assay validity of oxidative stress markers [1–3]. Blood leakage into saliva as a result of microinjury represents an important factor that is expected to influence the concentrations of markers of oxidative stress in saliva. The blood contamination in saliva is more common in individuals suffering from poor oral health and in patients with gingivitis or periodontitis. The concentrations of oxidative stress markers are typically several times higher in plasma than in saliva [4]. In patients with gingivitis blood leakage into saliva could artificially increase concentrations of salivary markers of oxidative stress. Recently, the effect of blood contamination on salivary concentrations of selected hormones was shown [5–7]. Despite the rising popularity of salivary oxidative stress analyses in patients with periodontal diseases, no reports have been published regarding the effect of blood contamination in saliva on concentrations of oxidative stress markers. The standardization of methodological processes is a key step before the implementation of salivary biomarkers for disease prediction and progression.

The aim of our study was to analyze the effect of artificial whole blood contamination and the effect of contamination with individual blood components (plasma, red blood cells, and hemoglobin) on salivary concentrations of markers of oxidative stress in healthy probands. In addition, the impact of blood contamination should be studied in a case-control study comparing the salivary markers of oxidative stress in patients with gingivitis and healthy controls after dental hygiene treatment.

## 2. Subjects and Methods

### 2.1. Participants

In study I 10 young periodontally healthy volunteers (5 females and 5 males) with an average age of 23.5 ± 1.9 years were enrolled. In study II a total of 22 subjects were enrolled in the dental ambulance in Bratislava, Slovakia. Twelve subjects were male patients with gingivitis with an average age of 35.3 ± 8.0 years and 10 male subjects were age-matched healthy controls with an average age of 38.2 ± 4.9 years. In study II subjects underwent an examination of their periodontal status using plaque index (PI) [8], sulcus bleeding index (SBI) [9], and bleeding on probing (BOP). All clinical measurements were performed by a single investigator (LB). Exclusion criteria in both studies were systematic diseases, acute illnesses, pregnancy, smoking, and former smoking. The studies were approved by the Ethics Committee of the Institute of Molecular Biomedicine, Comenius University, Bratislava, Slovakia. The clinical part of this study was performed according to the principles expressed in the Declaration of Helsinki. Written informed consent was obtained from each participating subject.

### 2.2. Design and Sampling

Whole unstimulated saliva samples were collected in the morning before eating. Collected saliva samples were stored at −20°C until analyses. On the day of testing, samples were brought to room temperature and centrifuged at 1000 g for 10 min and the supernatant was used for testing.

In study I saliva samples were artificially contaminated with blood. Samples of saliva were divided into aliquots. One aliquot from each individual was used as a control (no blood added). The remaining salivary aliquots were contaminated by venous blood and serially diluted to obtain saliva samples with the following concentrations of blood: 10%, 5%, 2.5%, 1%, 0.1%, 0.01%, and 0.001%. Similar to contamination of saliva with whole blood other aliquots were contaminated with plasma, red blood cells, or hemoglobin (Sigma Aldrich, Steinheim, Germany) ranging from 10% to 0.001%.

In study II a baseline saliva sample was collected from the participants. Dental hygiene treatment was performed by a dentist (LB). Saliva samples were collected again after treatment. Dental hygiene treatment was used as a model of blood leakage due to microinjury.

### 2.3. Biochemical Analysis of Oxidative and Carbonyl Stress Markers in Saliva

All reagents or chemicals used in our experiments were purchased from Sigma-Aldrich (Steinheim, Germany). Salivary advanced oxidation protein products (AOPP) as markers of protein oxidation were determined using a spectrophotometric method. Two hundred *μ*L of saliva was incubated with glacial acetic acid and the absorbance was read at 340 nm. Chloramine T with potassium iodide was used as calibrator [10]. The intra-assay and interassay variability are 6.6% and 12.4%, respectively.

Salivary advanced glycation end products (AGEs) as markers of carbonyl stress were measured using spectrofluorometric method. Saliva samples were diluted 10-fold with phosphate buffered saline (PBS, pH = 7.2) and measured at *λ*_ex._ = 370 nm, *λ*_em._ = 440 nm [11]. The specific fluorescence of AGEs was expressed in arbitrary units. The intra-assay and interassay variability are 8.9% and 10.5%, respectively.

Ferric reducing antioxidant power (FRAP), marker of antioxidant status, was determined according to Benzie and Strain [12]. The intra-assay and interassay variability are 1.7% and 9%, respectively. Briefly, prewarmed 37°C FRAP reagent (1 volume of 3 mol/L acetate buffer, pH 3.6 + 1 volume of 10 mmol/L 2,4,6-tripyridyl-S-triazine in 40 mmol/L HCl + 1 vol of 20 mmol/L FeCl_3_) was mixed with 20 *μ*L of saliva. Absorbance was read at 593 nm. Ferrous sulphate was used as standard in calibration curve.

Total antioxidant capacity (TAC), marker of antioxidant status, was measured using spectrophotometric method. Saliva was mixed with acetate buffer (pH = 5.8), incubated with 2,2′-azino-bis(3-ethylbenzthiazoline-6-sulphonic acid) and oxidized with hydrogen peroxide in acetate buffer (pH = 3.6). Absorbance was measured at 660 nm. Trolox was used as standard in calibration curve [13]. The intra-assay and interassay variability are 6.6% and 12.4%, respectively.

Total proteins were quantified using BCA protein assay kit (Sigma Aldrich, Steinheim, Germany). Briefly 10 *μ*L of saliva was mixed with BCA working reagent, incubated for 30 min at 37°C and measured at 562 nm. Concentrations of salivary oxidative stress markers were normalized to total proteins. All measurements were done on a Sapphire II instrument (Tecan, Grödig, Austria).

### 2.4. Statistical Analysis

Analysis was performed with XLStatistics 10.05.30 (Carr, R., XLent Works, Australia) and GraphPad Prism 5.03 (GraphPad Software, San Diego, California). In study I two-way repeated measures (RM) ANOVA was used to analyze oxidative stress markers in saliva artificially contaminated with blood. Based on the results from two-way RM ANOVA data from both genders were combined and analyzed using one-way RM ANOVA and Tukey's multiple comparison test. In study II the effect of microinjury on salivary markers of oxidative stress before and after dental hygiene was determined using Wilcoxon matched-pairs signed rank test for control and gingivitis group separately. Data are presented as mean + SD. Level *α* = 0.05 was chosen as a limit level of significance.

## 3. Results

### 3.1. Study I

**Figure 1:**
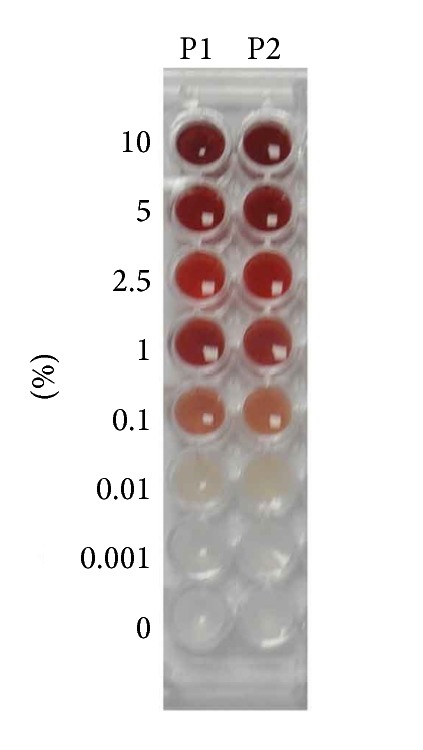
Saliva samples of two probands (columns P1 and P2) contaminated by venous blood with the following final concentrations of blood: 10%, 5%, 2.5%, 1%, 0.1%, 0.01%, 0.001%, and 0%. Note the visible discoloration of saliva with blood contamination from 0.1%.

**Figure 2:**
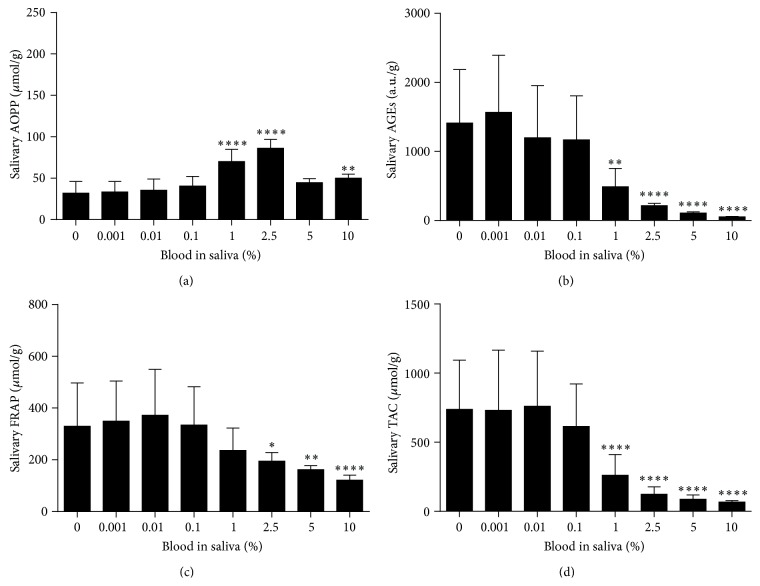
Oxidative stress and antioxidant status markers in saliva contaminated with blood. (a) Salivary AOPP concentrations, biomarker of oxidative damage to proteins. (b) Salivary AGEs concentrations, biomarker of carbonyl stress. (c) Salivary FRAP concentrations, biomarker of antioxidant status. (d) Salivary TAC concentrations, biomarker of antioxidant status. Data are presented as mean + SD; ^*∗∗∗∗*^ *p* < 0.0001, ^*∗∗*^ *p* < 0.01, and ^*∗*^ *p* < 0.05.

Saliva samples contaminated by whole blood with a final concentration of 0.1% blood and higher are visibly colored (Figure 1). The effect of two independent variables, gender and blood contamination on dependent variable, oxidative stress markers was analyzed using two-way RM ANOVA. Separate ANOVAs were used for whole blood, plasma, red blood cells, and hemoglobin contamination. Because no major effect of gender was observed, data from both genders were combined for further analyses (Table 1). One-way RM ANOVA and Tukey's multiple comparison test were used for further analyses. A significant bias in the measured concentrations of salivary oxidative stress markers was caused by 1% blood contamination in saliva (Figure 2). AOPP as a marker of protein oxidation was significantly higher in saliva contaminated with 1% and 2.5% venous blood by 118.7% and 168.5%, respectively (*q* = 13.13 and *q* = 18.63, *p* < 0.0001, Tukey's test, Figure 2(a)). In saliva samples contaminated with 5% and 10% blood AOPP concentrations were lower in comparison to samples contaminated with 2.5% blood (Figure 2(a)). Salivary carbonyl stress measured as AGEs concentrations decreased proportionally when blood was added to saliva at concentrations 1–10% (Figure 2(b)). Concentrations of antioxidant status markers FRAP and TAC also decreased in blood-contaminated saliva (Figures 2(c) and 2(d)).

**Figure 3:**
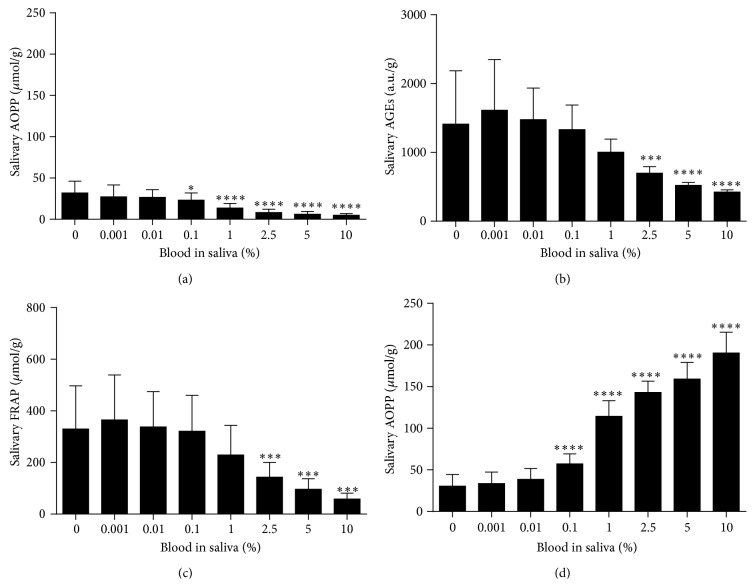
Oxidative stress and antioxidant status markers in saliva contaminated with plasma. (a) Salivary AOPP concentrations, biomarker of oxidative damage to proteins. (b) Salivary AGEs concentrations, biomarker of carbonyl stress. (c) Salivary FRAP concentrations, biomarker of antioxidant status. (d) Salivary TAC concentrations, biomarker of antioxidant status. Data are presented as mean + SD; ^*∗∗∗∗*^ *p* < 0.0001, ^*∗∗∗*^ *p* < 0.001, ^*∗∗*^ *p* < 0.01, and ^*∗*^ *p* < 0.05.

**Figure 4:**
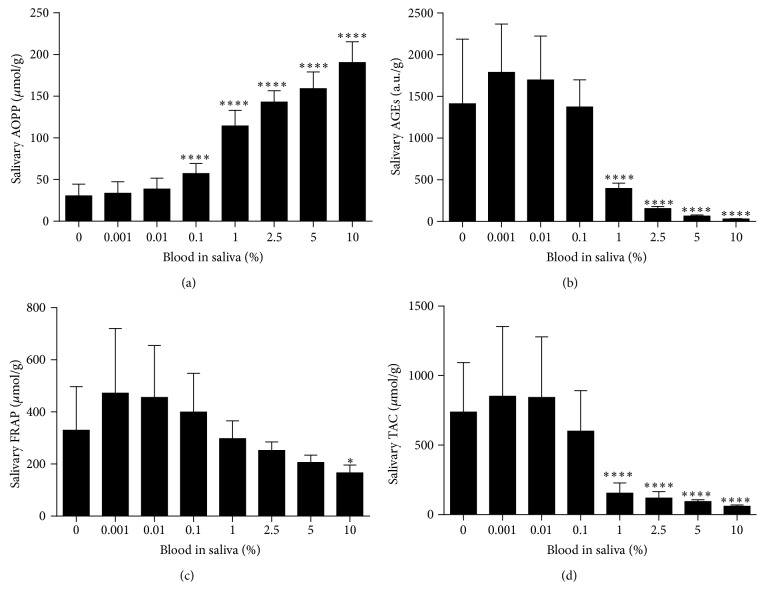
Oxidative stress and antioxidant status markers in saliva contaminated with red blood cells (RBC). (a) Salivary AOPP concentrations, biomarker of oxidative damage to proteins. (b) Salivary AGEs concentrations, biomarker of carbonyl stress. (c) Salivary FRAP concentrations, biomarker of antioxidant status. (d) Salivary TAC concentrations, biomarker of antioxidant status. Data are presented as mean + SD; ^*∗∗∗∗*^ *p* < 0.0001 and ^*∗*^ *p* < 0.05.

**Figure 5:**
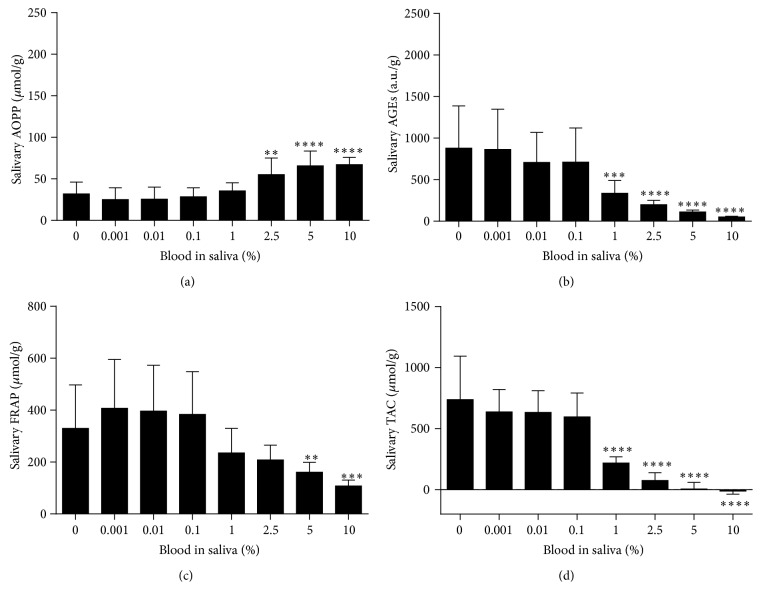
Oxidative stress and antioxidant status markers in saliva contaminated with hemoglobin. (a) Salivary AOPP concentrations, biomarker of oxidative damage to proteins. (b) Salivary AGEs concentrations, biomarker of carbonyl stress. (c) Salivary FRAP concentrations, biomarker of antioxidant status. (d) Salivary TAC concentrations, biomarker of antioxidant status. Data are presented as mean + SD; ^*∗∗∗∗*^ *p* < 0.0001, ^*∗∗∗*^ *p* < 0.001, and ^*∗∗*^ *p* < 0.01.

To determine which blood component is responsible for changes in measured salivary markers, the impact of plasma, red blood cells, and hemoglobin was studied. Salivary AOPP concentrations were decreased proportionally in the presence of 0.1–10% plasma contamination in saliva by 27.3–85.0% (Figure 3(a)). Salivary AOPP concentrations were increased in the presence of 0.1–10% red blood cells by 80.4–493.3% (Figure 4(a)). A similar trend was observed after addition of 2.5–10% hemoglobin to saliva with increased AOPP concentrations by 72.1–109.4% (Figure 5(a)). Salivary AGEs were increased in the presence of 2.5–10% plasma in saliva by 50.6–69.9% (Figure 3(b)). Addition of 1–10% red blood cells or hemoglobin resulted in comparable changes (Figures 4(b) and 5(b)). Concentrations of antioxidant status markers FRAP and TAC were decreased in saliva when plasma (Figures 3(c) and 3(d)), red blood cells (Figures 4(c) and 4(d)), or hemoglobin (Figures 5(c) and 5(d)) were added.

### 3.2. Study II

**Figure 6:**
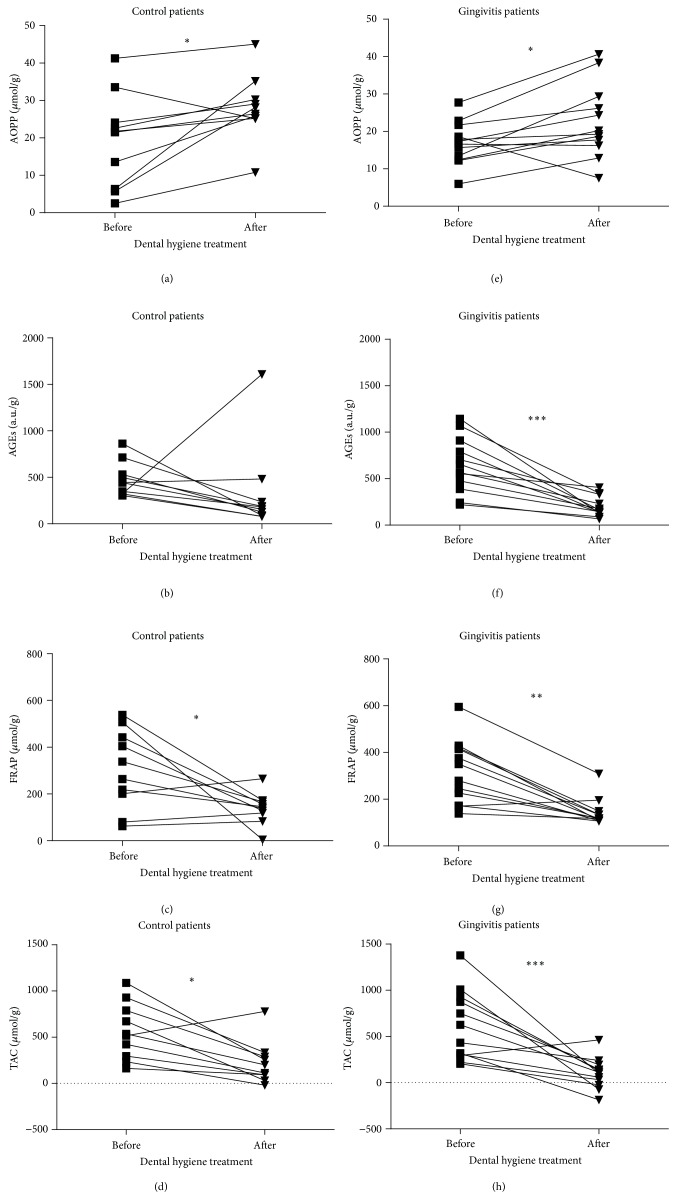
The effect of blood leakage after dental hygiene treatment on markers of oxidative stress and antioxidant status in saliva of control (a–d) and gingivitis (e–h) patients. (a, e) Salivary AOPP concentrations, biomarker of oxidative damage to proteins. (b, f) Salivary AGEs concentrations, biomarker of carbonyl stress. (c, g) Salivary FRAP concentrations, biomarker of antioxidant status. (d, h) Salivary TAC concentrations, biomarker of antioxidant status. ^*∗∗∗*^ *p* < 0.001, ^*∗∗*^ *p* < 0.01, and ^*∗*^ *p* < 0.05.

To study the effect of blood contamination in a real clinical situation the impact of microinjury in gingivitis and age-matched healthy control patients was modeled. Clinical parameters of both study groups are summarized in Table 2. Clinical parameters were significantly worse in the gingivitis group compared with the control group (Table 2). Dental hygiene as model of microinjury caused blood leakage in both patients groups. The effect of microinjury on salivary markers of oxidative stress before and after dental hygiene was determined using Wilcoxon matched-pairs signed rank test for control and gingivitis group separately. Salivary AOPP concentrations were increased in 9 out of 10 control probands and in 10 out of 12 gingivitis patients after microinjury (Figures 6(a) and 6(e)). Salivary AGEs concentrations were decreased in 9 out of 10 control probands and in 12 out of 12 gingivitis patients (Figures 6(b) and 6(f)). Salivary FRAP concentrations were decreased in 7 out of 10 control probands and in 11 out of 12 gingivitis patients after treatment (Figures 6(c) and 6(g)). Salivary TAC concentrations were decreased in 9 out of 10 in control probands and in 11 out of 12 gingivitis patients after microinjury (Figures 6(d) and 6(h)).

## 4. Discussion

Schwartz and Granger reported that blood components in saliva invisible to the eye have the potential to bias salivary analytes and the control of blood contamination in saliva was suggested. Transferrin enzymatic immunoassay was designed for quantitative monitoring of blood contamination [14]. It was shown that concentrations of testosterone, dehydroepiandrosterone (DHEA), and cortisol are increased in saliva samples artificially contaminated with blood [14]. On the other hand, microinjury of the oral cavity did not change DHEA or cortisol and increased salivary testosterone [6]. Despite the rising number of studies analyzing oxidative stress in saliva, no reports have been published regarding the effect of occult blood contamination on concentrations of salivary markers of oxidative stress.

Four markers of oxidative stress and antioxidant status were analyzed in our study. Advanced oxidation protein products (AOPP), a novel oxidative stress biomarker was discovered in the plasma of uremic patients in 1996 [10]. Recently AOPP was suggested as part of the nonenzymatic antioxidant system of plasma proteome and oxidized fibrinogen was indicated as key molecule responsible for human plasma AOPP reactivity [15]. Advanced glycation end products (AGEs), marker of carbonyl stress, are developed during the reaction of protein amino groups with reactive carbonyl compounds [16]. Ferric reducing antioxidant power (FRAP) assay indirectly reflects the total antioxidant capacity of the sample [12, 17]. Total antioxidant capacity (TAC) assay developed by Erel is direct measurement method for total antioxidants [13]. Our results have shown that most of the measured salivary markers of oxidative stress and antioxidant status are biased in the presence of 1% blood contamination in saliva. However, as shown in results section saliva contaminated with 1% blood is visually discolored. Blood-contaminated saliva samples can be easily excluded from the analyses. Thus, at least for the markers analyzed in this study, there is no need to use salivary transferrin assay for the monitoring of blood contamination.

To determine which blood component is responsible for changes in measured salivary markers, the impact of plasma, red blood cells, and hemoglobin was studied. Based on our results increased AOPP concentrations in blood-contaminated saliva can be explained by the presence of red blood cells and hemoglobin in saliva. Hemoglobin in saliva probably interferes with the colorimetric AOPP assay and artificially increases the AOPP concentrations. AOPP concentrations in plasma of healthy probands were reported as 3 times as high as AOPP concentrations in saliva [15]; we have therefore expected increased AOPP concentrations in saliva contaminated with plasma. But an opposite trend was observed when plasma was spiked into saliva and decreased salivary AOPP was detected in the presence of 0.1–10% plasma. When plasma is spiked into saliva, the concentration of total proteins is increased. In our study decreased concentrations of measured salivary markers after plasma addition into saliva could be caused by normalization to the increased total proteins. Based on our results hemoglobin in saliva can mask the detection of AGEs and lead to underestimation of AGEs in saliva. Plasma addition into saliva led to decreased salivary AGEs concentrations. Salivary FRAP and TAC concentrations were decreased in the presence of RBC, hemoglobin, and plasma contamination in saliva. Decreased concentrations of measured markers in saliva contaminated with plasma could be as in the case of AOPP assay caused by normalization to total proteins.

The effect of blood contamination in saliva on concentrations of salivary markers of oxidative stress was not studied in real clinical situation in the past. Concerning that blood contamination in saliva is common in patients with gingivitis the impact of microinjury was modeled in this study group and age-matched healthy controls. The results were similar to saliva artificially contaminated with blood. AOPP concentrations were increased after microinjury in the visual presence of blood in saliva. AOPP concentrations were increased in both control and gingivitis group after dental hygiene treatment. AOPP concentrations were not different between control and gingivitis patients before dental hygiene treatment. Also other measured salivary markers followed the trend observed in saliva artificially contaminated with blood. Salivary AGEs concentrations and also antioxidant markers TAC and FRAP were decreased after dental hygiene treatment due to presence of blood in saliva. Our results have confirmed the concern that dental hygiene treatment could bias the concentrations of oxidative stress markers in saliva. Based on our results we recommend the saliva collection before dental hygiene treatment or clinical examination of oral cavity.

## 5. Conclusions

Salivary oxidative stress concentrations are significantly influenced by 1% blood contamination in saliva. Saliva samples with 1% blood contamination are visibly colored and it is possible to easily exclude such contaminated samples from further salivary oxidative stress analyses. Microinjury to the periodontium caused blood leakage into saliva in both gingivitis and control group and biased concentrations of oxidative stress markers in saliva. For the purpose of salivary oxidative stress analyses saliva samples should be collected before dental hygiene treatment or clinical examination of the oral cavity.

## Figures and Tables

**Table 1:** The effect of gender and blood contamination on oxidative stress markers was analyzed using two-way RM ANOVA. Separate ANOVAs were computed for whole blood, plasma, red blood cells (RBC), and hemoglobin contamination.

	Two-way RM ANOVA *F*	*p* value	*p* value summary
*Salivary AOPP *			
Blood contamination			
Effect of blood	49.86	<0.0001	*∗∗∗∗*
Effect of gender	0.51	0.49	ns
Plasma contamination			
Effect of plasma	33.59	<0.0001	*∗∗∗∗*
Effect of gender	3.68	0.09	ns
RBC contamination			
Effect of RBC	296.3	<0.0001	*∗∗∗∗*
Effect of gender	0.20	0.66	ns
Hemoglobin contamination			
Effect of hemoglobin	28.31	<0.0001	*∗∗∗∗*
Effect of gender	1.36	0.28	ns

*Salivary AGEs *			
Blood contamination			
Effect of blood	21.39	<0.0001	*∗∗∗∗*
Effect of gender	5.42	0.0483	*∗*
Plasma contamination			
Effect of plasma	20.75	<0.0001	*∗∗∗∗*
Effect of gender	4.48	0.07	ns
RBC contamination			
Effect of RBC	68.02	<0.0001	*∗∗∗∗*
Effect of gender	5.25	0.05	ns
Hemoglobin contamination			
Effect of hemoglobin	19.17	<0.0001	*∗∗∗∗*
Effect of gender	6.52	0.0340	*∗*

*Salivary FRAS *			
Blood contamination			
Effect of blood	12.00	<0.0001	*∗∗∗∗*
Effect of gender	3.878*e* ^−008^	0.99	ns
Plasma contamination			
Effect of plasma	23.59	<0.0001	*∗∗∗∗*
Effect of gender	0.27	0.62	ns
RBC contamination			
Effect of RBC	9.772	<0.0001	*∗∗∗∗*
Effect of gender	0.18	0.68	ns
Hemoglobin contamination			
Effect of hemoglobin	18.32	<0.0001	*∗∗∗∗*
Effect of gender	0.8376	0.39	ns

*Salivary TAC *			
Blood contamination			
Effect of blood	24.31	<0.0001	*∗∗∗∗*
Effect of gender	0.20	0.67	ns
Plasma contamination			
Effect of plasma	33.02	<0.0001	*∗∗∗∗*
Effect of gender	0.51	0.49	ns
RBC contamination			
Effect of RBC	24.37	<0.0001	*∗∗∗∗*
Effect of gender	0.25	0.63	ns
Hemoglobin contamination			
Effect of hemoglobin	43.80	<0.0001	*∗∗∗∗*
Effect of gender	0.15	0.71	ns

AOPP, advanced oxidation protein products; AGEs, advanced glycation end products; FRAP, ferric reducing antioxidant power; TAC, total antioxidant capacity; ns, nonsignificant.

^*∗*^ *p* < 0.05; ^*∗∗∗∗*^ *p* < 0.0001.

**Table 2:** Comparison of clinical parameters between control and gingivitis patients.

Clinical parameter	Group		Unpaired *t*-test *t*	*p* value	*p* value summary
	Control	Gingivitis			
	(*n* = 10)	(*n* = 12)			
BOP (%)	18.52 ± 4.69	74.32 ± 13.00	12.9	<0.0001	*∗∗∗∗*
SBI (score)	0.40 ± 0.12	1.72 ± 0.33	11.9	<0.0001	*∗∗∗∗*
PI (score)	0.58 ± 0.15	1.14 ± 0.44	3.9	0.0010	*∗∗*

BOP, bleeding on probing; SBI, sulcus bleeding index; PI, plaque index. Data are presented as mean ± SD.

^*∗∗*^ *p* < 0.01; ^*∗∗∗∗*^ *p* < 0.0001.
